# Satiety-enhancing placebo intervention decreases selective attention to food cues

**DOI:** 10.3389/fpsyt.2024.1472532

**Published:** 2024-12-19

**Authors:** Marina Lanz, Verena Hoffmann, Karin Meissner

**Affiliations:** ^1^ Institute of Medical Psychology, Faculty of Medicine, Ludwig-Maximilians-University Munich, Munich, Germany; ^2^ Department for Pediatric Medicine, Schwabing Hospital, Munich, Germany; ^3^ Division of Health Promotion, Coburg University of Applied Sciences and Arts, Coburg, Germany

**Keywords:** placebo effect, expectation, attentional bias, selective attention, appetite, satiety, food craving, visual probe task

## Abstract

**Background:**

As placebo interventions could influence appetite and satiety in first studies, they are a promising tool for the future treatment of obesity. Furthermore, individuals with heightened body weight show increased selective attention for food cues. This study aimed to investigate whether placebo induced changes of appetite and satiety can affect attention allocation and to examine correlating factors.

**Methods:**

In a double-blind design, 63 healthy participants were randomized into one of three groups: the enhanced appetite placebo group, the enhanced satiety placebo group, or the control group. Appetite and satiety were induced by administering a placebo capsule along with a group specific expectancy manipulation. One hour later, participants performed a visual probe task to measure attentional bias by comparing reaction times for different conditions. Correlations between reaction times and subjective hunger and satiety ratings, as well as current food craving and plasma ghrelin levels, were explored.

**Results:**

The induction of attentional bias toward non-food stimuli was successful in women in the enhanced satiety placebo group but not in the enhanced appetite placebo group. Women of the enhanced satiety placebo group showed significantly higher reaction times for food cues compared to non-food cues. Across conditions, reaction times were associated with subjective hunger ratings and current food craving in women. No attentional bias was induced in men in either placebo group.

**Conclusion:**

Placebo-induced satiety inhibited attention allocation toward food in healthy women, potentially mediated by reduced hunger and food craving. Placebo effects on satiety could thus be demonstrated on a highly complex cognitive process.

## 1 Introduction

Overweight and obesity are major cardiovascular risk factors and continue to pose a growing global health challenge. As of 2022, 43% of adults worldwide were classified as overweight, and 16% as obese (1). Although increased energy intake and reduced physical activity are primary contributors to elevated body weight (2), recent studies have also focused on the neurobiological and cognitive mechanisms regulating appetite and satiety (3, 4) in search of novel therapeutic strategies. Modifying attention to food cues has emerged as a promising approach for addressing dysregulated attentional processes related to appetite.

Attentional bias arises from various attention-regulation mechanisms that enable the brain to prioritize processing of relevant stimuli, helping meet immediate needs as efficiently as possible (5). Early research on attentional bias primarily focused on threat-related stimuli (6, 7), yet biases have since been observed for a range of stimuli types (8, 9). Food-related stimuli, often perceived positively, may inherently induce attentional bias (10), particularly when addressing an immediate need like hunger (8). For example, in studies using the dot probe task, hungry participants demonstrated heightened attentional bias toward food-related words (e.g., chocolate, honey) over neutral words, as evidenced by faster reaction times (11). Additionally, elevated attentional bias toward food cues has been observed in overweight and obese individuals compared to those of normal weight, suggesting a dysregulated attentional process in obesity similar to that seen in individuals with substance use disorders (12, 13). Functional MRI studies further support this, showing a positive correlation between body mass index (BMI) and activation in brain regions associated with attention when exposed to food cues (14).

Appetite and satiety are closely tied to mental states such as craving (15) and expectations. For instance, studies show that participants preferred Coke more when it was branded (16), and perceived taste ratings for cheese and yogurt were lower when labeled as fat-reduced (17). Expectations also influence physiological appetite regulation, including the release of the appetite-stimulating hormone ghrelin. In a study by Crum and colleagues, participants were given an identical milkshake described as either “high-calorie, indulgent” or “low-calorie, sensible,” and blood ghrelin levels were measured before and after consumption. Participants consuming the “indulgent” milkshake displayed a significantly steeper decline in ghrelin levels, suggesting a stronger sense of satiety compared to the “sensible” milkshake (18). Positive expectations, a major component of the placebo effect, could therefore be harnessed to modulate appetite and satiety. Recent research further shows that beliefs about a hunger-altering placebo intervention influence medial prefrontal cortex activation and even impact later food choices (19). Additional evidence comes from studies demonstrating that when the expected satiety from a previous meal is higher, calorie intake at the next meal is lower (20), underscoring the powerful effect of expectations on eating behavior. Finally, a recent systematic review provides initial evidence suggesting that placebo interventions can promote weight loss in adults (21).

In this study, we integrated insights from placebo and attention research to examine whether placebo-induced changes in appetite and satiety can influence attentional bias toward food cues. Within a larger trial investigating placebo effects on appetite, satiety, and their objective markers, participants were administered a placebo capsule with instructions that it would either stimulate appetite or increase satiety (22). Later, a subgroup of these participants completed a visual probe task, one of the most established paradigms for measuring attentional bias (23). We hypothesized that placebo-induced appetite would heighten attention to food cues, evidenced by faster reaction times for food stimuli relative to neutral stimuli, while placebo-induced satiety would reduce attention to food cues. Additionally, we explored both behavioral and physiological factors that might moderate attentional bias in our analyses.

## 2 Material and methods

### 2.1 Participants

Healthy participants aged 18-40 years with normal body weight (BMI 19-25kg/m^2^) were included in the main study, within which this substudy was embedded. Exclusion criteria comprised pregnancy or breastfeeding, smoking, alcohol or drug abuse, food allergies, regularly intake of medication (except contraceptives), acute or chronic disease, history of psychiatric disease, surgery within the last four weeks prior to participation, elevated fasting blood glucose levels (>100mg/dl), and clinically relevant anxiety or depression scores [score >7 in at least one subscale of the Hospital Anxiety and Depression Scale (HADS) (24)]. Recruitment was conducted through university mailing lists and flyers. All participants provided written informed consent and received 45€ as compensation.

### 2.2 Study design

**Figure 1:**
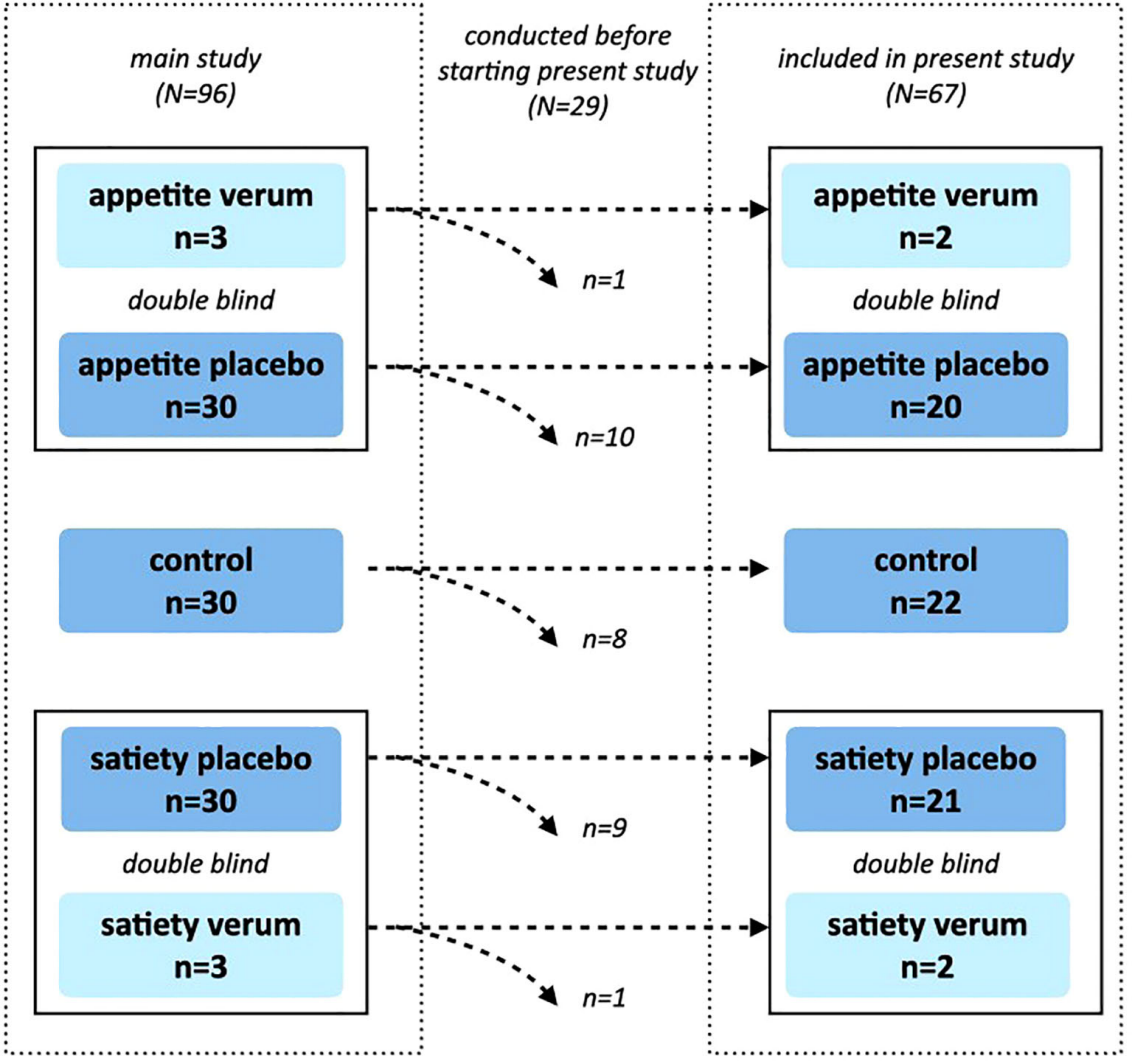
Study Design. The main study was designed as a randomized, double-blind, controlled trial, in which 96 participants were randomized into one of five groups (control, appetite placebo, appetite verum, satiety placebo, satiety verum). A total of 29 measurements had already been conducted before the start of this substudy. Verum groups were included only for double-blinding and were not analyzed further. Randomization was maintained, which led to slightly different group sizes within the substudy.

This study was conducted at the Institute of Medical Psychology at Ludwig-Maximilians-University Munich, Germany, and was nested within a larger double-blinded randomized controlled trial investigating the neurobiological mechanisms of placebo interventions on appetite and satiety [for details, see (22)]. In the main study, 90 participants were randomly allocated to one of three groups stratified by sex: enhanced appetite placebo, enhanced satiety placebo, or control. For ethical reasons and to ensure double-blinding, six additional participants were randomly allocated to the enhanced appetite verum and enhanced satiety verum groups (**Figure 1**). Since this substudy started later than the main study, only 67 out of the 96 study participants from the main study were included. The original study protocol and the amendment describing this substudy were approved by the ethics committee of the Medical Faculty, Ludwig-Maximilians-University Munich, Germany (approval number 650-15).

### 2.3 Randomization and blinding

As detailed in (22), sequentially numbered and sealed envelopes were prepared by a person not directly involved in the experiment, using a computer-generated randomization list. Each envelope contained a capsule along with information about the type of intervention (either appetite-enhancing, satiety-enhancing, or control). To ensure double-blinding, neither the participant nor the experimenter knew whether the capsule in the appetite- or satiety-enhancing intervention contained a placebo or an active ingredient.

### 2.4 Experimental procedure

The experimental procedure of the main experiment is described in detail in Hoffmann and colleagues (22). In brief, each participant underwent a single experimental session starting at 8 a.m. after fasting for 10 to 12 hours. Finger blood samples to measure blood glucose levels were taken using a BG Star device (Sanofi-Aventis, Hannover, Germany). Blood samples to assess ghrelin levels were repeatedly drawn from a peripheral intravenous catheter flushed with saline. After placing electrodes to monitor the electrocardiogram and the electrogastrogram, participants completed the “Hospital Anxiety and Depression Scale” [HADS (24)] and the “Food Craving Questionnaire - Trait” [FCQ-T (25, 26)], rated their current levels of hunger and satiety on 100 mm visual analogue scales (VAS), and assessed their current food craving using the “Food Craving Questionnaire – State” [FCQ-S (25)]. Thereafter, the first ghrelin blood sample was taken. The experimenter then opened the sealed envelope, performed the verbal expectancy manipulation according to group allocation, and the participant swallowed the provided capsule with 100 ml of mineral water. After each of two consecutive 30 min resting periods, hunger and satiety ratings were assessed, and ghrelin blood samples were collected. Participants were then asked to complete the FCQ-S (25) again. Following a brief instruction on the visual probe task (VPT) by the experimenter, participants performed the VPT while the experimenter left the room to minimize distraction. After completing the VPT, participants in the appetite- and satiety-enhancing intervention groups were asked to guess their allocation to either the verum or placebo group.

### 2.5 Interventions

Lactose (Heirler Cenovis GmbH, Radolfzell, Germany) was used as the inert ingredient for the control group and placebo groups, while an alginate complex (CM3 Alginat Kapseln, Easyway GmbH, Monheim, Germany) served as the satiety-enhancing active ingredient, and a bitter herb extract (Appetit-Anreger, Zirkulin Naturheilmittel GmbH, Bremen) was used as the appetite-enhancing active ingredient. In the appetite-enhancing and satiety-enhancing intervention groups, participants were informed that they would receive a capsule containing a real treatment or a placebo. Participants in the control group were told they would receive a capsule with an inert substance [for details, see (22)].

### 2.6 Visual probe task

The VPT was designed using “Visual Studio, Framework.net 4.5.2” in C# and performed on a fast PC. Participants were seated 500 mm from a 400 mm screen. One hundred pictures were paired, with five pairs used for a brief instruction. The remaining 45 pairs included either two pictures of neutral objects (15 pairs) or one picture of appetizing food paired with one neutral object (30 pairs). All pictures were sourced from the “food.pics database” (27) and screened for similar complexion, object size, brightness, and contrast within each pair. Additionally, food pictures were included only if they had a calorie density between 250-500 kcal/100g and total calories greater than 250 kcal per picture, along with high craving and palatability scores in the database’s internal ratings. Participants rated the 30 food types depicted in the selected pictures for personal craving prior to the experimental session day. Only the 15 highest-rated food picture pairs were used to create individualized VPTs for each participant, enhancing the internal reliability of the task by selecting the most attractive stimuli (28).

A total of 120 reaction times (RTs) under three different conditions were measured. Each trial began with a fixation cross displayed for 500 ms in the center of the screen. Two hundred and fifty milliseconds after its disappearance, two pictures were displayed for 100 ms each, positioned 60 mm to the left and right of the center, respectively. Immediately after the pictures disappeared, a dot appeared at the former position of one of the pictures. Participants were instructed to detect the dot as quickly as possible and then press a button (“A” for left or “L” for right) to confirm detection. The time between the appearance of the dot and the button press was recorded as RT. After a variable interval of 300 ms to 2000 ms, the next trial started with the display of the fixation cross (29, 30).

**Figure 2:**
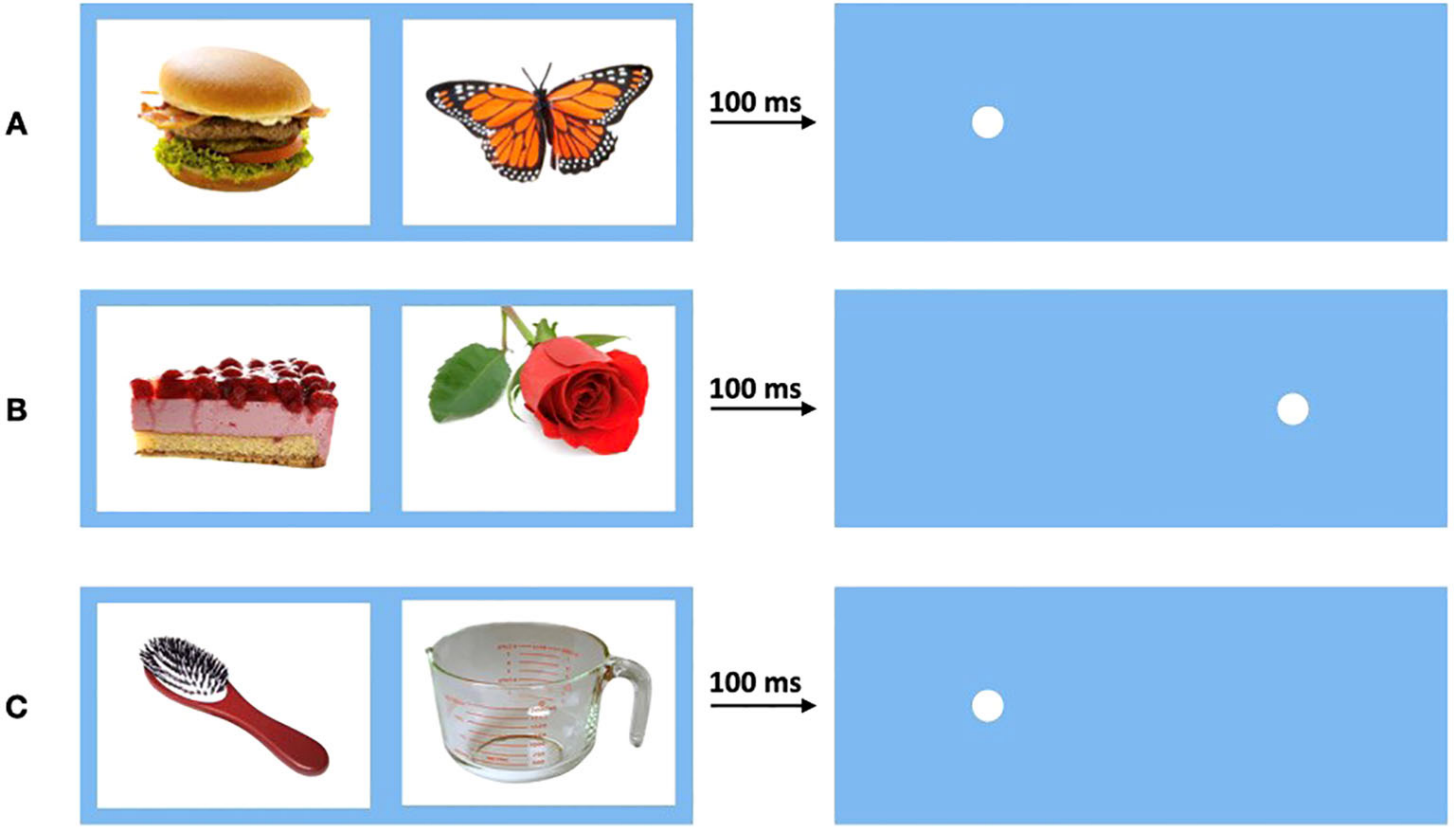
Conditions of the visual probe task. Pictures were displayed for 100 ms before disappearing, then dot appears. **(A)** Condition congruent: One neutral and one food picture. Dot appears behind food picture. **(B)** Condition incongruent: One neutral and one food picture. Dot appears behind neutral picture. **(C)** Condition baseline: Two neutral pictures. Dot appears behind one of neutral pictures.

The three conditions were defined as congruent (30 trials per participant), incongruent (30 trials per participant), and baseline (60 trials per participant). Trials featuring two neutral objects were classified as baseline, measuring the participant’s individual ordinary RT. If one picture was a neutral object and the other a food object, the trial was considered congruent if the dot appeared at the location of the food picture and incongruent if it appeared at the location of the neutral picture (**Figure 2**).

### 2.7 Outcome parameters

#### 2.7.1 Reaction times of visual probe task

As primary outcome parameters, the mean RTs and standard deviations (SD) for each condition (*M*_baseline_, *M*_congruent_, *M*_incongruent_) were calculated from the 120 measured RTs per participant. Before calculation, outliers were excluded following a previously established three-stage process similar to other outlier exclusion procedures in studies using the VPT (29, 31). First, all trials with errors (e.g., pressing the wrong button) or trials with RTs <100 ms or >2000 ms were removed, as they clearly do not reflect the true RTs. Second, all trials differing by more than 3.5 SD from the participant’s condition-specific mean RT were excluded. Third, all trials differing by more than 2.5 SD from the condition-specific mean RT of the entire sample were excluded, as they might reflect atypical situations (e.g., lack of understanding of the test, poor concentration).

In the VPT, lower mean RT for the congruent condition (*M*_congruent_) compared to the baseline condition (*M*_baseline_) indicates a higher orienting toward food stimuli, while a higher mean RT for the incongruent condition (*M*_incongruent_) compared to M_baseline_ indicates delayed disengagement from food stimuli. Conversely, a higher mean RT for the congruent condition (*M*_congruent_) compared to the baseline condition (*M*_baseline_) indicates delayed disengagement from non-food stimuli, while a lower mean RT for the incongruent condition (*M*_incongruent_) compared to *M*_baseline_ indicates enhanced orienting toward non-food stimuli.

#### 2.7.2 Behavioral parameters

A 100 mm VAS was used for repeated measurements of subjective hunger and satiety ratings, ranging from “not at all hungry/full” to “extremely hungry/full”. Additionally, a dichotomous scale was administered to determine whether participants believed they had received a placebo or an active treatment.

#### 2.7.3 Questionnaires

The Food Craving Questionnaire – State (FCQ-S; 25) and the short version of the Food Craving Questionnaire – Trait (FCQ-T; 26) were employed to assess general food craving and to measure current food craving at multiple time points. Both the state and trait measures of food craving have been shown to enhance selective attention to food cues (15). Furthermore, the Hospital Anxiety and Depression Scale (HADS) was utilized to screen for elevated levels of anxiety and depression, following the suggested cut-off values [(24); see above]. Additionally, participants were asked to provide their body weight and height for the calculation of the BMI.

#### 2.7.4 Plasma ghrelin

To measure plasma ghrelin levels, blood samples were taken at three different time points into commercially available EDTA tubes (2.7ml) prepared with 54 µl of 4mM 4-(2-aminoethyl)benzenesulfonyl fluoride hydrochloride (AEBSF) (32). The samples were immediately stored on ice after collection until they were centrifuged within 60 minutes for 10 minutes at 3,000g and 4°C. Two subsamples of 500 µl per blood sample were transferred to Eppendorf tubes prepared with 100 µl of 1 mM HCl and gently mixed before being stored at -70°C until final analyses. Plasma ghrelin levels (pg/ml) were measured in duplicate, following the protocol, using the Human Ghrelin (total) ELISA Kit (Catalogue number EZGRT-89K, Merck Millipore, Darmstadt, Germany).

### 2.8 Statistical analyses

Before analysis, all data were assessed for normality using skewness, kurtosis, and the Kolmogorov-Smirnov test. All continuous outcome parameters met the normality assumption. To account for individual differences in RTs, a mixed analysis of covariance (ANCOVA) was employed to explore the RTs of the VPT. In this analysis, *M*_baseline_ served as a covariate, while condition-specific mean RTs (“condition”) were treated as a within-subjects factor. The factors “group” (enhanced appetite placebo, enhanced satiety placebo, control) and “sex” were included as between-subjects factors. To directly compare *M*_congruent_ and *M*_incongruent_ with *M*_baseline_, two-tailed t-tests were used. Bonferroni corrections for multiple comparisons were applied where appropriate to control for the increased risk of Type I errors. Dichotomous parameters were analyzed using the chi-square test.

Pearson correlation coefficients were calculated to assess possible relationships between subjective states of hunger, satiety, and food craving, individual RTs, ghrelin levels, and general food craving. Participants from both placebo groups (enhanced appetite placebo and enhanced satiety placebo) and the control group were included, as an association between appetite regulation and RTs was expected in the entire sample. To account for participants’ individual ordinary RTs, difference scores (Δ) were calculated and entered into the correlational analyses as follows: for the congruent condition (Δ*M*_congruent_ = *M*_congruent_ – *M*_baseline_) and for the incongruent condition (Δ*M*_incongruent_ = *M*_incongruent_ – *M*_baseline_). To account for baseline differences in hunger, satiety, and food craving ratings as well as ghrelin levels, Δ scores were computed and entered into the correlational analyses as follows: for hunger (ΔHunger (VAS) = VAS_hunger before VPT_ – VAS_hunger baseline_), for satiety (ΔSatiety (VAS) = VAS_satiety before VPT_ – VAS_satiety baseline_), for current food craving (ΔFood craving (FCQ-S) = FCQ-S_before VPT_ – FCQ-S_baseline_), and for ghrelin levels (ΔGhrelin = Ghrelin_beforeVPT_ – Ghrelin_baseline_).

All statistical analyses were performed using SPSS (Version 25), and a significance level of *p* ≤ 0.05 was assumed.

## 3 Results

### 3.1 Participants

Out of 240 individuals who responded to the recruitment efforts for the main study, 113 were screened for eligibility. Seventeen participants were excluded before the experimental session (3 did not meet inclusion criteria, 12 did not provide informed consent, and one did not show up). Additionally, one participant was retrospectively excluded due to elevated fasting blood glucose levels and was replaced with a new participant. In total, 96 participants were included and completed the experimental session. Twenty-nine of these were tested before commencing this substudy. Four of the remaining participants received a verum treatment and were therefore excluded from further analyses. Consequently, data from 63 participants (31 men, 32 women) were included in the following analyses (**Figure 1**).

The study groups were comparable at baseline in terms of demographic, behavioral, and humoral parameters, as well as general food craving scores, and anxiety and depression scores (**Table 1**). The mean age of participants was 23.6 years (SD = 2.9), and the mean BMI was 21.6 (SD = 1.8).

**Table 1:** Group characteristics at baseline.

	Control (n=22) mean ± SD	Appetite Placebo (n=20) mean ± SD	Satiety Placebo (n=21) mean ± SD	p-value
**Sex**	12 women 10 men	10 women 10 men	10 women 11 men	
**Age (years)**	23.2 ± 3	24 ± 2.6	23.6 ± 3.1	0.666
**BMI (kg/m^2^)**	22 ± 1.9	21.5 ± 1.9	21.4 ± 1.6	0.484
**Hunger (VAS)**	5.4 ± 2.7	5.0 ± 2.8	5.6 ± 2.3	0.731
**Satiety (VAS)**	2.2 ± 1.9	3.8 ± 3.1	2.4 ± 2.4	0.106
**Food Craving State (FCQ-S)**	26.1 ± 6.9	23.1 ± 5.6	26.3 ± 5.0	0.144
**Blood Glucose (mg/dl)**	95.5 ± 6.7	94.6 ± 7.9	97.2 ± 9.7	0.607
**Ghrelin Level (pg/ml)**	391 ± 119	422 ± 175	368 ± 124	0.510
**Food Craving Trait (FCQ-T)**	40.3 ± 12.8	34.8 ± 9.7	35.1 ± 10.4	0.196
**Anxiety (HADS)**	3.0 ± 2.1	3.2 ± 2.5	3.2 ± 1.5	0.909
**Depression (HADS)**	1.6 ± 1.9	1.4 ± 1.6	1.9 ± 2.1	0.742

SD, Standard Deviation; VAS, Visual Analogue Scale; FCQ-S, Food Craving Questionnaire – State; FCQ-T, Food Craving Questionnaire – Trait; HADS, Hospital Anxiety and Depression Scale.Bold values indicate significant p-values (p<0.05).

### 3.2 Reaction times in visual probe task

**Figure 3:**
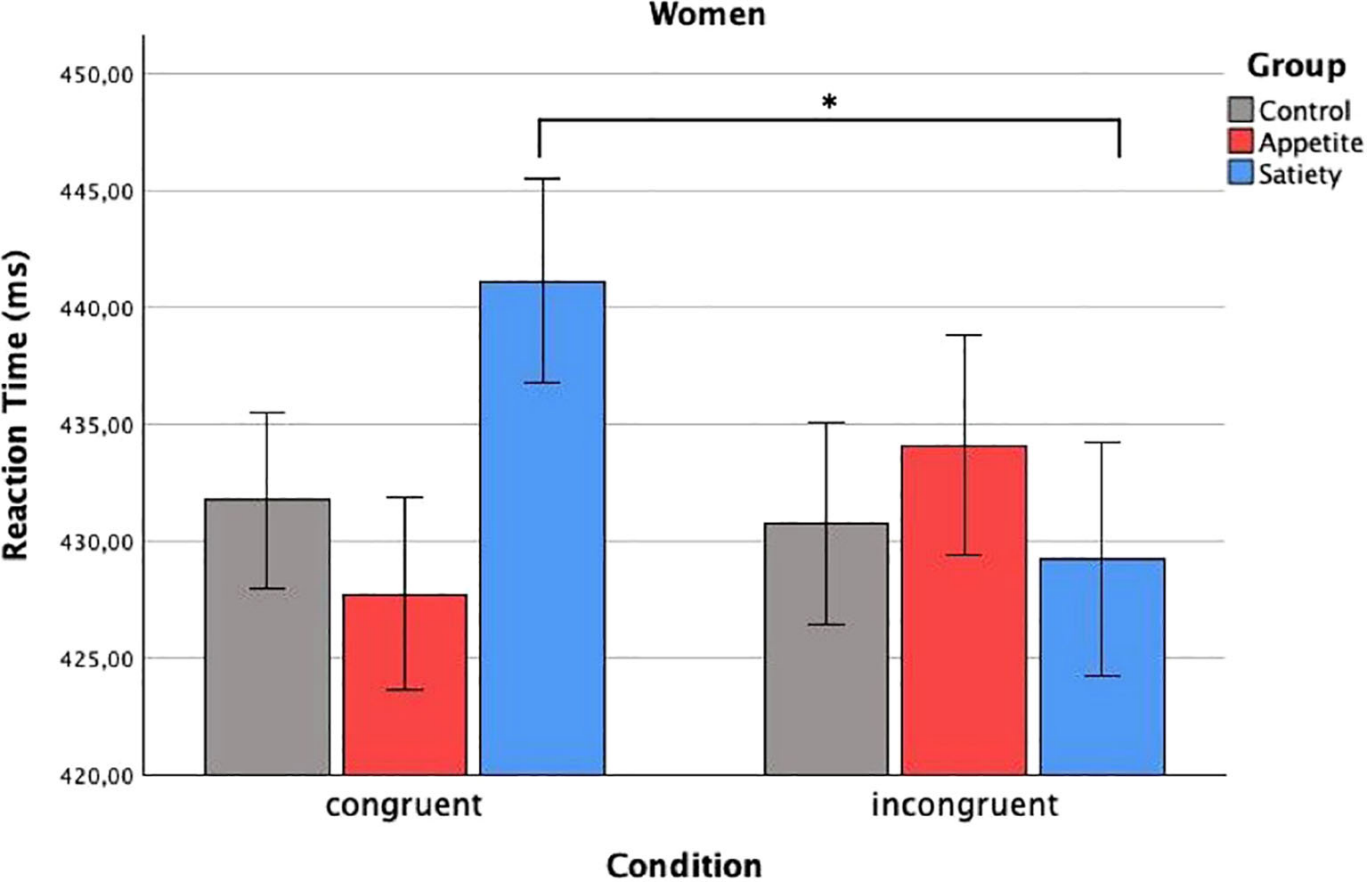
Reaction times in women. Baseline-corrected reaction times (ms) in women according to condition and group (estimated means ± SD). Significant difference of reaction time to congruent and incongruent condition in the group placebo-satiety. *p<0.05.

**Figure 4:**
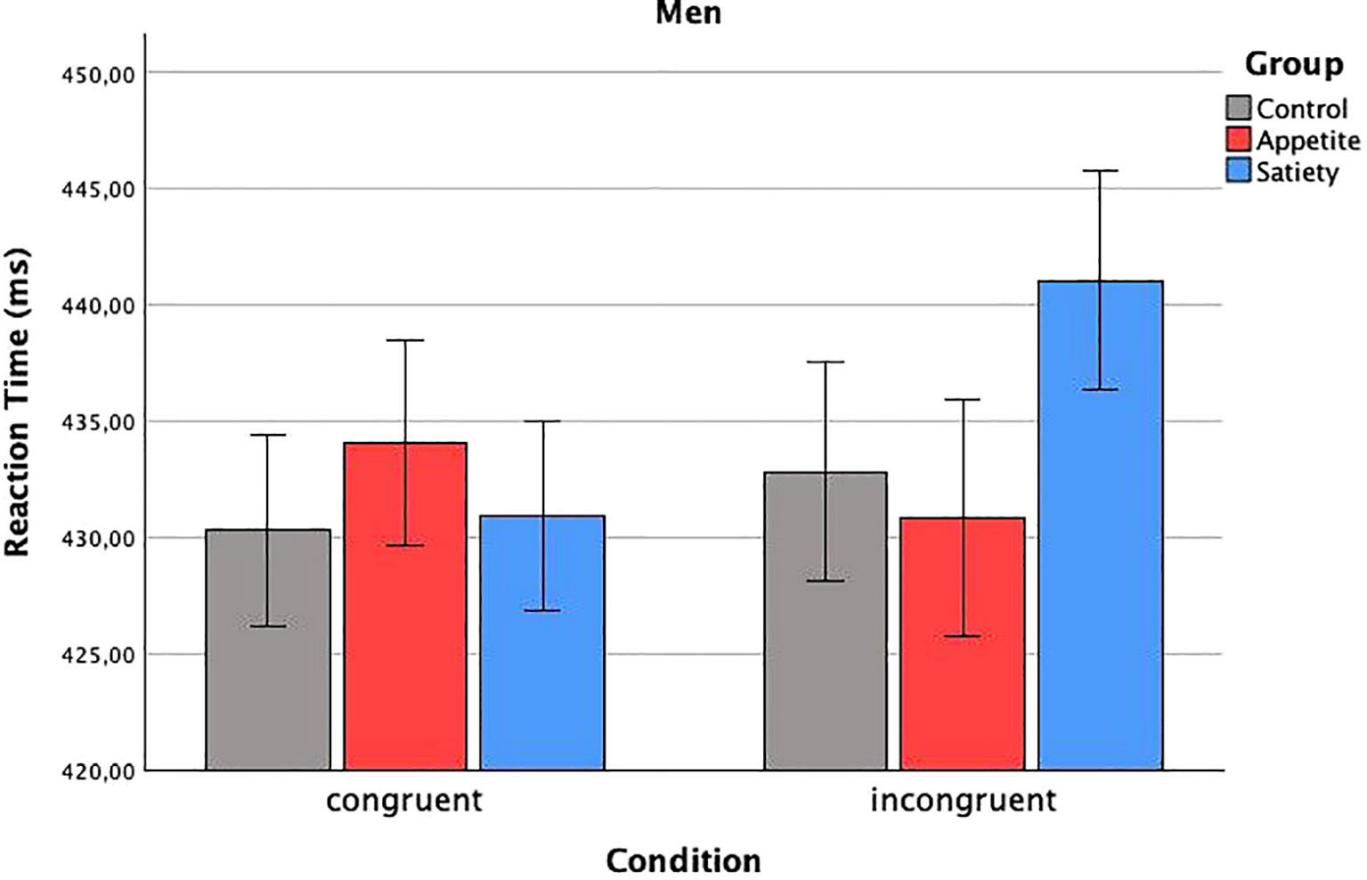
Reaction times in men. Baseline-corrected reaction times (ms) in men according to condition and group (estimated means ± SD). No significant difference.

In total, 4.3% of the data were removed during the prescribed outlier exclusion process (2.1% in the first step, 0.6% in the second step, and 1.6% in the third step, the latter leading to exclusion of the VPT data of one participant in the enhanced appetite placebo group). The mixed ANCOVA revealed a significant three-way interaction (*F*_condition x group x sex_(2, 55) = 4.19, p = 0.020) (**Table 2**). Bonferroni-corrected two-way ANCOVAs, conducted separately for the placebo groups, showed a significant interaction between “condition” and “sex” in the enhanced satiety placebo group (*F*_condition x sex_(1, 18) = 6.14, p = 0.046), but not in the enhanced appetite placebo group (*F*_condition x sex_(1, 16) = 2.27, p = 0.304). Further Bonferroni-adjusted *post hoc* tests for the enhanced satiety placebo group revealed *M*_congruent_ was significantly higher than *M*_incongruent_ in women (*F*_condition_(1,8) = 8.1, p = 0.044) (**Figure 3**), while this was not the case for men (*F*_condition_(1,9) = 0.004, p = 1) (**Figure 4**). To investigate whether this effect in women could be attributed to delayed disengagement from or enhanced orienting toward the food cues, two separate Bonferroni-corrected t-tests comparing *M*_congruent_ and *M*_incongruent_ with *M*_baseline_ in female participants from the enhanced satiety placebo group were performed. Neither test was significant (*M*_congruent_: t_(9)_ = -1.236, p = 0.496, *M*_incongruent_: t_(9)_ = 0.687, p = 1).

**Table 2:** Means of M_congruent_ and M_incongruent_ (controlled for M_baseline_) by group and sex.

	Control		Appetite Placebo		Satiety Placebo	
	Female (n=12) mean ± SE	Male (n=10) mean ± SE	Female (n=10) mean ± SE	Male (n=9) mean ± SE	Female (n=10) mean ± SE	Male (n=11) mean ± SE
**M_congruent_ (ms)**	431.7 ± 3.8	430.3 ± 4.1	427.7 ± 4.1	434.1 ± 4.4	441.1 ± 4.4	430.9 ± 4.1
**M_incongruent_ (ms)**	430.8 ± 4.3	432.8 ± 4.7	434.1 ± 4.7	430.8 ± 5.1	429.2 ± 5.1	441.0 ± 4.7

SE, Standard Error.

### 3.3 Correlations between reaction times and hunger, satiety, and food craving ratings

Pearson correlation coefficients were calculated separately for men and women. In women, significant correlations emerged between Δ*M*_incongruent_ and ΔHunger ratings (r = 0.356, p = 0.046) as well as ΔFood craving (r = 0.389, p = 0.028), but not with ΔSatiety ratings (r = -0.334, p = 0.062). In contrast, Δ*M*_congruent,_ was not related to ΔHunger ratings, ΔSatiety ratings, or ΔFood craving in women. All analyses were repeated for men, and no significant correlations were found (**Table 3**).

**Table 3:** Pearson correlation coefficients between ΔM_congruent_ and ΔM_incongruent_ (controlled for M_baseline_) and behavioral ratings (ΔHunger, ΔSatiety, ΔFood craving, controlled for baseline levels), humoral measurements (ΔGhrelin, controlled for baseline levels), and general food craving.

	Women		Men	
	Δ M_congruent_	Δ M_incongruent_	Δ M_congruent_	Δ M_incongruent_
ΔHunger (VAS)	n=32 r = 0.075 p = 0.685	**n=32** **r = 0.356** p = 0.046*	n=30 r = -0.136 p = 0.473	n=30 r = -0.262 p = 0.162
ΔSatiety (VAS)	n=32 r = 0.049 p = 0.790	n=32 r = -0.334 p = 0.062	n=30 r = 0.101 p = 0.595	n=30 r = 0.093 p = 0.626
ΔFood craving (FCQ-S)	n=32 r = 0.052 p = 0.778	**n=32** **r = 0.389** p = 0.028*	n=30 r = -0.084 p = 0.658	n=30 r = -0.236 p = 0.210
ΔGhrelin (pg/ml)	n=29 r = 0.296 p = 0.119	n=29 r = 0.127 p = 0.510	n=26 r = 0.193 p = 0.344	n=26 r = -0.048 p = 0.817
General food craving (FCQ-T)	n=32 r = 0.196 p = 0.282	n=32 r = 0.052 p = 0.778	n=30 r = 0.020 p = 0.915	n=30 r = -0.225 p = 0.231

VAS, Visual Analogue Scale; FCQ-S, Food Craving Questionnaire – State; FCQ-T, Food Craving Questionnaire – Trait; *p<0.05.Bold values indicate significant p-values (p<0.05).

### 3.4 Correlations between reaction times and ghrelin levels

Neither Δ*M*_congruent_ nor Δ*M*_incongruent_ correlated with ΔGhrelin in either sex (**Table 3**).

### 3.5 Correlations between reaction times and general food craving

Neither Δ*M*_congruent_ nor Δ*M*_incongruent_ correlated with general food craving (FCQ-T) in either sex (**Table 3**).

### 3.6 Treatment guesses

In the satiety placebo group, nine out of 21 participants (42.9%) believed they received an active treatment, while only one out of 20 participants (5%) in the appetite placebo group thought the same. A significant group difference was observed (χ^2^ = 7.96, p = 0.005). In total, 4 women (20%) and 6 men (29%) in the placebo groups guessed they had received an active ingredient. There was no significant difference between sexes (χ^2^ = 0.41, p = 0.523).

## 4 Discussion

To our knowledge, this is the first study to investigate attentional bias toward food cues following placebo-induced appetite and satiety in healthy men and women. The results partially confirm our hypothesis that placebo-induced appetite and satiety would affect selective attention to food stimuli. Notably, we demonstrated for the first time that women receiving the placebo intervention to enhance satiety exhibited longer reaction times for food cues compared to neutral stimuli, indicating reduced attention to food cues after expectancy manipulation. Conversely, the placebo intervention to enhance appetite did not affect attention allocation to food cues neither in women nor in men.

Our findings align with several studies demonstrating decreased selective attention to food cues following satiety induction. For example, a study observed a decreased percentage of fixations and reduced viewing time of food pictures in an eye movement paradigm among women with weight concerns after satiety was induced using a placebo pill paired with a verbal suggestion (33). Another study using the VPT revealed an attentional bias toward two different food types in a hungry state. After consumption of one food type until sated, a significant decrease of selective attention to that food was detected, while no such decrease was observed for the uneaten food (34). These studies, in conjunction with our findings, suggest that selective attention adjusts rapidly and even indicate that attention is drawn away from stimuli addressing satisfied needs.

Interestingly, despite successful satiety induction in both sexes (22), no attentional bias could be detected in men from the enhanced satiety placebo group. Treatment guesses also did not differ between men and women, refuting the idea that men’s potentially higher skepticism accounts for this discrepancy. Recent studies suggest that sex significantly influences the relationship between eating behavior and visual attention to food cues; for instance, emotional eating correlates with visual attention only in women (35). Additionally, women tend to focus more on low-calorie, low-fat foods (36, 37) but are more likely to consume sweet snacks when experiencing stress (38), whereas men generally pay more attention to both sweet and savory high-calorie foods (37). In this study, the predominance of high-calorie food images could partly explain the observed gender difference. While placebo-induced satiety may reduce selective attention to high-calorie foods in women, it may not counteract men’s naturally higher attention toward such foods.

The sex-specific pattern in attentional bias, despite similar reductions in satiety for both sexes, is also consistent with findings from placebo research. While men and women reported comparable placebo effects on visceral pain and nausea, their neurobiological correlates differed (39, 40). Specifically, the insular cortex showed varying activation patterns, with placebo effects in men involving neural down-regulation of the insular cortex and related interoceptive experiences, such as pain or nausea. Conversely, placebo effects in women were more closely associated with prefrontal cortex activity (40). According to a recent functional MRI study, placebo-induced satiety in women may result from altered perceptual attentional filtering, where the perceived tastiness of food becomes less relevant under decreased hunger conditions (19).

Contrary to our expectations and prior studies (11, 29), we did not observe an attentional bias for food cues in the enhanced appetite placebo group. The most likely explanation for this finding is that the appetite-enhancing placebo intervention did not successfully induce increased hunger in our study (22). Notably, significantly more participants believed they had received an active treatment in the satiety-enhancing intervention group, suggesting that the placebo intervention was more credible in this group than in the appetite-enhancing intervention group.

In our female participants, decreased selective attention to food cues was correlated with changes in hunger ratings and current food craving. A recent meta-analysis similarly provided evidence for a positive correlation between selective attention to food cues and both current food craving and subjective hunger (41). Notably, general food craving did not correlate with attentional bias for food cues, neither in previous research (42) nor in our study. In addition, the correlation of hunger and food cravings with *M*_incongruent_ but not *M*_congruent_ suggests that the altered attentional bias may result from enhanced orienting toward non-food stimuli rather than delayed disengagement from non-food stimuli.

In this study, we used a cue exposure duration of 100 ms, as prior studies on food attentional bias, especially when using the VPT, indicated this duration as beneficial (7, 29). Very short exposure durations (<250 ms) serve as subliminal exposures that appear to engage early attentional processes such as enhanced orienting. Support for this interpretation comes from an eye-tracking study showing that, in a fed state, normal-weight individuals tend to direct their initial gaze to the non-food picture, indicating an initial orienting response to the non-food stimulus (31). Furthermore, Potthoff and colleagues found a higher percentage of fixation on non-food images after placebo-induced satiety, pointing in a similar direction (33).

Contrary to our expectations, no correlation between the appetite-enhancing gut hormone ghrelin and attentional bias was detected. Notably, ghrelin levels remained unchanged in the enhanced satiety placebo group (22). These findings support the conclusion that placebo-induced satiation is not mediated by ghrelin. Rather, verbally induced expectations may lead to a top-down modulation of satiety that operates independently of humoral mechanisms. Further evidence for this interpretation comes from a recent study showing that, although expectations of increased satiety reduced calorie intake in subsequent meals and throughout the day, no effect on ghrelin levels was observed (20). However, further research should examine whether other peptides involved in appetite regulation, such as cholecystokinin, glucagon-like peptide 1, and peptide YY, may play a role in expectation-enhanced satiety (43).

Several limitations of this study should be noted. First, because we analyzed men and women separately, each group size was limited to 10 to 12 participants. Future studies may benefit from reducing the number of intervention groups or increasing the sample size. Second, this study examined only healthy, normal-weight participants. Including overweight or obese individuals would allow for investigation into whether placebo interventions can alter the dysregulated attentional processes associated with obesity (12). Third, we used expectancy manipulation, rather than conditioning, to induce placebo effects on satiety and appetite. Conditioning, which is known to engage unconscious processes and produce longer-lasting placebo responses (44, 45), could be advantageous for addressing long-term outcomes such as body weight.

We propose three key areas for further research. First, the potential of deregulating visual attention as a therapeutic tool should be explored for individuals with overweight and obesity. Ideally, this research would integrate multimodal strategies that combine approaches to achieve rapid and sustainable weight loss, ultimately reducing cardiovascular risk. Second, future longitudinal studies could use conditioning to generate longer-lasting placebo effects. Notably, a recent study using an expectancy-based placebo application demonstrated a decrease in appetite only during the first two days of a seven-day follow-up period (46). Third, the neurobiological mechanisms underlying placebo-induced satiety and the subsequent reduction in selective attention to food cues, as well as potential sex-specific differences, warrant further investigation. In this context, functional MRI could be a promising tool, especially for examining the involvement of the brain’s reward system, which plays a crucial role in food-related behaviors and can override physiological appetite control (47).

In conclusion, the results of the present study demonstrate that placebo-induced satiety inhibits attention allocation toward food cues in healthy women, presumably mediated by decreased subjective hunger and food craving. Attention allocation, as a largely unconscious process, verifies the placebo effect on satiety also at an objective level. Our findings align with growing evidence that placebo interventions, particularly those utilizing expectancy effects, can significantly influence food craving and related behaviors. Rodríguez-Martín and colleagues (48) demonstrated that expectancy-based placebo interventions were effective in controlling food craving and intrusive food-related thoughts over time. This suggests that cognitive factors, such as the belief in a treatment’s efficacy, may modulate attention to and desire for food independently of physiological hunger signals. In our study, similar top-down processes likely contributed to the observed placebo effects on attentional bias and satiety in women. These findings support the potential of placebo-based cognitive interventions not only in normal-weight individuals but also as a feasible adjunct for managing food craving in overweight and obese populations, offering a promising avenue for further research on placebo-induced modulation of eating behavior.

## Data Availability

The raw data supporting the conclusions of this article will be made available by the authors, without undue reservation.
